# AAV9-Mediated TNPO3 Overexpression in the Heart Rescues RBM20 Cardiomyopathy in Mice

**DOI:** 10.1161/CIRCULATIONAHA.124.072766

**Published:** 2025-09-09

**Authors:** Julia Kornienko, Laura Schraft, Kai Fenzl, Maarten M.G. van den Hoogenhof, Lars M. Steinmetz

**Affiliations:** 1 Genome Biology Unit, European Molecular Biology Laboratory (EMBL), Heidelberg, Germany (J.K., L.S., K.F., L.M.S.).; 2 DZHK (German Centre for Cardiovascular Research), partner site Heidelberg/Mannheim, Heidelberg, Germany (J.K., K.F., M.M.G.v.d.H., L.M.S.).; 3 Institute of Experimental Cardiology, University Hospital Heidelberg, Germany (M.M.G.v.d.H.).; 4 Department of Genetics, Stanford University School of Medicine, CA (L.M.S.).; 5 Stanford Genome Technology Center, Palo Alto, CA (L.M.S.).

**Keywords:** cardiomyopathies, genes, genetic therapy, mice, precision medicine

The *RBM20* gene encodes a cardiac-enriched alternative splicing factor that regulates the differential isoform expression of *TTN*, *CAMK2D*, and other transcripts essential for heart function.^1^ Sequence variations in *RBM20* cause aggressive dilated cardiomyopathy with early disease onset. The majority of identified disease-causing variants are heterozygous missense sequence variations that cluster in a conserved stretch of 6 amino acids (PRSRSP) located within the arginine/serine-rich (RS) domain.^1^ These variants lead to protein mislocalization into cytoplasmic phase-separated ribonucleoprotein granules, which results in a toxic gain of function.^2^ Mislocalized, variant RBM20 (RNA-binding motif protein 20) binds to 3′ untranslated regions of transcripts in the cytoplasm, colocalizes with processing bodies, and disrupts cellular contractility more than an *RBM20* knock-out.^3^ We previously identified TNPO3 (transportin 3) as a direct nuclear importer of RBM20 in humans.^4^ We showed that 2 disease-causing RS domain variants (P633L and R634Q) disrupt the RBM20–TNPO3 interaction proportionally to the severity of their mislocalization. Whereas the R634Q variant has a strong impact on TNPO3 interaction, resulting in severe mislocalization, the P633L variant displays a milder phenotype. We showed that partial nuclear relocalization of these variants in induced pluripotent stem cell–derived cardiomyocytes by TNPO3 overexpression proportionally restored *TTN* splicing, whereas full nuclear relocalization rescued the splicing dysfunction to levels observed in wild-type (WT) cells.^4^ Here, we provide the first proof of principle that overexpression of TNPO3 in vivo can rescue mis-splicing and cardiac dysfunction in RBM20-P635L^+/+^ and RBM20-R636Q^+^^/-^ mice (corresponding to P633L and R634Q variants in humans).

We injected 4-week-old RBM20-WT, RBM20-P635L^+/+^, RBM20-R636Q^+/-^, and RBM20-R636Q^+/+^ mice^5^ with AAV9 (adeno-associated virus serotype 9) vectors (10^12^ viral genomes) delivering either a nontargeting control (pCAG-iCre-T2A-eGFP [*Ctr*]) or murine *Tnpo3* cDNA (pCMV-mm*Tnpo3* [*Tnpo3*]) and analyzed them 12 weeks later (Figure [A]). We did not include P635L^+/-^ mice because they do not exhibit a heart failure phenotype.^5^ The treatment led to an ≈5-fold increase of median *Tnpo3* expression compared with endogenous levels (Figure [B]). We found that *Tnpo3* overexpression restored the ejection fraction and cardiac volume of P635L^+/+^ and R636Q^+/-^ mice over time to WT levels (Figure [C–E]). *Tnpo3*-treated WT and R636Q^+/+^ mice did not display significant changes in comparison with phosphate-buffered saline- or *Ctr*-injected mice (Figure [C] and [D]), indicating no negative impact of *Ctr* and *Tnpo3* treatments on cardiac function of healthy and dilated cardiomyopathy (DCM) mice. We measured the expression of *Ttn* and *Camk2d* isoforms, which are alternative splicing events dependent on functional RBM20.^1,4,5^ Their splicing was restored upon *Tnpo3* overexpression in P635L^+/+^ and R636Q^+/-^ mice to a level observed in WT mice (Figure [F] and [G]). Although the treatment of R636Q^+/+^ mice partially increased splicing function, the levels of rescue did not reach the WT level (Figure [F] and [G]), in agreement with the echocardiographic data (Figure [C–E]). This is likely because the R636Q substitution leads to a stronger disruption of RBM20-TNPO3 interaction, making restoration of nuclear import less efficient than for the P635L variant.^4^ Nevertheless, because all RS domain *RBM20* variants identified to date are heterozygous, the findings from the R636Q^+/-^ model are more relevant to patients.

**Figure.**
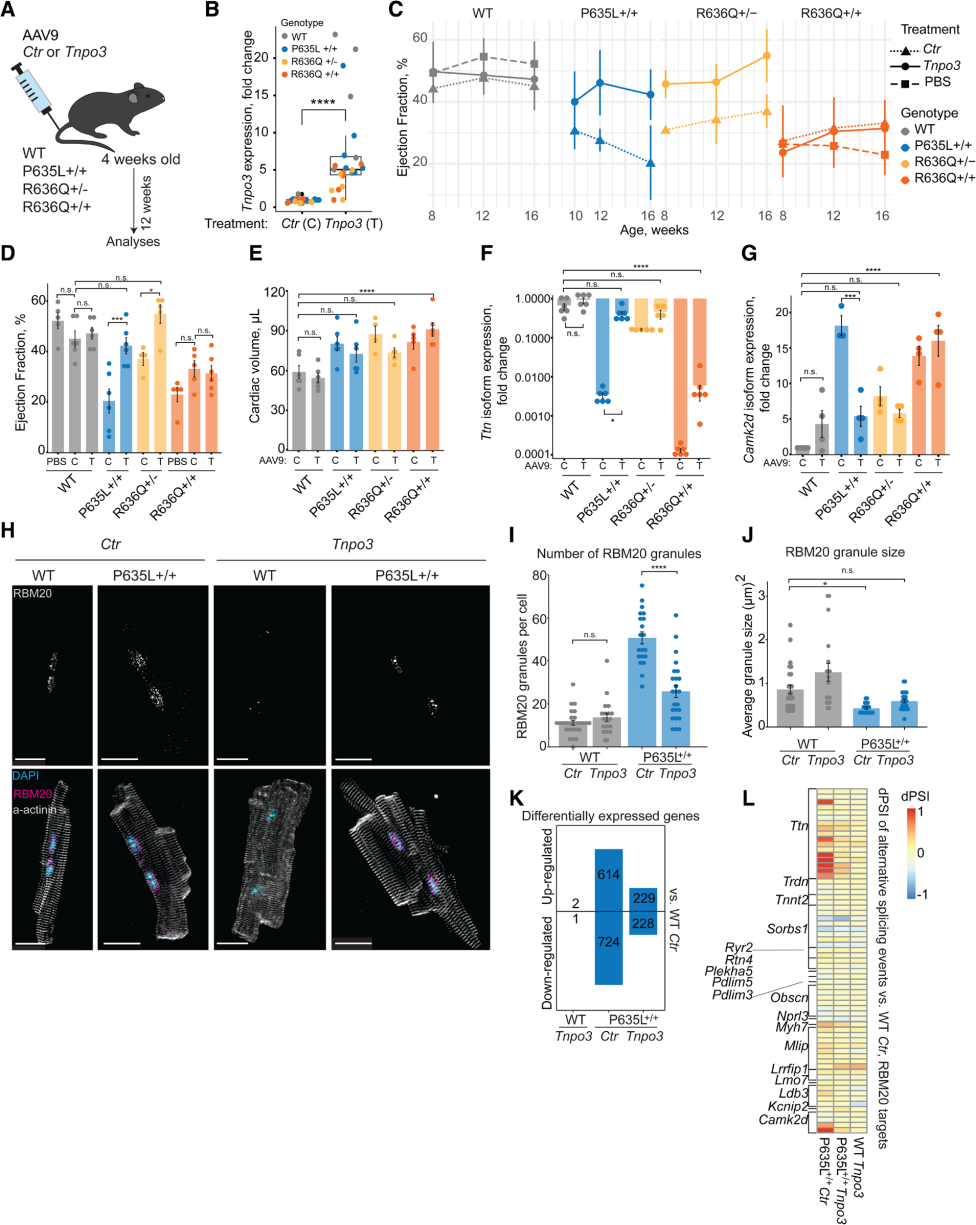
***Tnpo3* overexpression in vivo rescues the RBM20–dilated cardiomyopathy phenotype. A**, Schematic representation of the experiment timeline. **B**, Quantitative polymerase chain reaction analysis of *Tnpo3* expression in the left ventricle of RBM20-WT, RBM20-P635L^+/+^, RBM20-R636Q^+^^/-^, and RBM20-R636Q^+/+^ mice treated with either *Ctr* (C) or *Tnpo3* (T, 3 months after treatment; n=5 or 6 mice per group). **C**, Ejection fraction time course (n=5 or 6 mice per group). **D**, Ejection fraction and **E**, cardiac volume (diastole) 3 months after the treatment with Phosphate-buffered saline, *Ctr*, or *Tnpo3*, calculated on the basis of echocardiographic data of RBM20-WT, RBM20-P635L^+/+^, RBM20-R636Q^+/-^, and RBM20-R636Q^+/+^ mice (n=5 or 6 mice per group). **F**, Quantitative polymerase chain reaction analysis of *Ttn* spliced-out and **G**, *Camk2d* (*Camk2dA* and *Camk2d4*, combined) isoform expression in the left ventricle of RBM20-WT, RBM20-P635L^+/+^, RBM20-R636Q^+/-^, and RBM20-R636Q^+/+^ mice treated with either *Ctr* or *Tnpo3*. Data are normalized to *Gapdh* and displayed as fold change versus one of the RBM20-WT *Ctr* mice (3 months after treatment; n=3–6 mice per group). **H**, Representative microscopic images of isolated primary cardiomyocytes from RBM20-WT or RBM20-P635L^+/+^ mice treated with either *Ctr* or *Tnpo3.* Scale bar, 20 µm. **I**, RBM20-containing granule number and **J**, average RBM20 granule size quantified per cell using AggreCount plugin for Fiji. Each dot corresponds to 1 isolated primary cardiomyocyte (n=2 mice per group). **K**, Number of differentially expressed genes (absolute value of log2[fold change]>1 and *P*_adjusted_<0.05, DeSeq2), up- or downregulated compared with RBM20-WT *Ctr* mice (n=4 mice per group). **L**, Average (for 4 replicates) δ percentage of spliced-in (dPSI) values versus RBM20-WT *Ctr* mice for all alternative splicing events in the core RBM20 targets^1^ detected in all the pairwise comparisons (rMATS). Positive dPSI values (red) indicate that a single exon was more included in the sample compared with the RBM20-WT *Ctr* mice; negative values (blue) indicate that the exon was more excluded (n=4 mice per group). **B**, **D** through **G**, **I**, and **J**, 1-way ANOVA with 2-tailed Tukey honestly significant difference posttest was used for quantifying statistical significance. *P<0.05, **P<0.01, ***P<0.001, ****P<0.0001. ANOVA indicates Analysis of Variance; and WT, wild type.

To analyze how *Tnpo3* overexpression affects RBM20 granules, global gene expression, and alternative splicing, we focused on RBM20-WT and RBM20-P635L^+/+^ animals. We chose the P635L^+/+^ model rather than the R636Q^+/-^ model because it showed a stronger DCM phenotype and would thus provide a more conservative test for rescue. Indeed, upon *Tnpo3* overexpression, RBM20 in P635L^+/+^ mice could relocalize to the nucleus and form 2 characteristic^1,3^ nuclear foci (Figure [H]), supporting the splicing rescue data. *Tnpo3* treatment reduced the total number of RBM20-containing granules (Figure [I]) and increased the average granule size (Figure [J]) to similar levels of WT mice. Differential gene expression analysis in comparison with WT-*Ctr* mice showed that *Tnpo3* overexpression reduced the total number of differentially expressed genes in P635L^+/+^ mice (Figure [K]). Only 3 genes were differentially expressed between WT-*Ctr* and WT-*Tnpo3* mice (Figure [K]), with *Tnpo3* being the most significantly altered among them. This suggests that *Tnpo3* overexpression alone does not cause substantial changes in the gene expression profile. We further examined the alternative splicing of core RBM20 targets^1^ by calculating the average PSI values for RBM20 target exons. We compared these PSI values with those in WT-*Ctr* mice, thereby calculating δ-PSI (Figure [L]). *Tnpo3* overexpression in P635L^+/+^ mice restored alternative splicing of the majority of RBM20 target exons to levels seen in WT-*Ctr*, but did not noticeably affect WT mice (Figure [L]).

These results provide the first in vivo validation that *Tnpo3* overexpression restores alternative splicing and cardiac dysfunction in 2 patient-relevant RBM20-DCM models with no observed adverse effects on WT mice. Our findings serve as the first proof-of-principle that restoring nuclear localization of RBM20 RS-domain variants can rescue the alternative splicing deficiency and the cardiac pump function defect associated with DCM. This discovery provides a foundation for developing future therapies for DCM patients with mislocalizing *RBM20* variants by targeting nuclear relocalization of RBM20.

The primary limitation of the study was that absence of AAV9-control effects on cardiac function was experimentally verified only in WT and R636Q^+/+^ mice, whereas for other genotypes, this lack of effect was inferred from previous work.^5^

## ARTICLE INFORMATION

### Acknowledgments

The authors thank Marta Rodriguez-Martinez for project discussions; Markus Grosch and Sandra Clauder-Muenster for their work on generation and characterization of the mouse lines; European Molecular Biology Laboratory (EMBL) Laboratory Animal Resources for maintenance of the mouse lines and support, particularly Frank Diego Montoya Castillo and Alessandro Grassi for performing mouse injections, organ harvesting, and support, and the head of EMBL Laboratory Animal Resources, Ernesto de la Cueva, for help with experimental planning; the EMBL Gene Editing and Virus Facility for services; Adriana Castillo for producing, purifying, and titrating the AAV9 virus; the EMBL Genomics Core Facility for next-generation sequencing services; and the EMBL Advanced Light Microscopy Facility for support.

### Sources of Funding

This work was funded by the Deutsche Forschungsgemeinschaft (DFG, German Research Foundation) – SFB1550 – Project ID 464424253: Collaborative Research Center 1550 (CRC1550) "Molecular Circuits of Heart Disease". Dr Fenzl is supported by a research fellowship from the EMBL Interdisciplinary Postdoc (EIPOD) Programme under Marie Curie Cofund Actions MSCA-COFUND-FP (grant agreement 847543).

### Disclosures

Dr Steinmetz is a cofounder and shareholder of Sophia Genetics. Drs Kornienko, Fenzl, and Steinmetz filed an invention disclosure describing TNPO3 and restoring nuclear localization of RBM20 variants discussed in this article (US provisional patent application 63/452 252, filed March 15, 2023).
